# Patient-specific and healthcare real-world costs of atrial fibrillation in individuals treated with direct oral anticoagulant agents or warfarin

**DOI:** 10.1186/s12913-021-07125-5

**Published:** 2021-12-03

**Authors:** Mikko Pyykönen, Miika Linna, Markku Tykkyläinen, Eric Delmelle, Tiina Laatikainen

**Affiliations:** 1 Department of Geographical and Historical Studies, University of Eastern Finland, P.O. Box 111, 80101 Joensuu, Finland; 2 Department of Industrial Engineering and Management, Aalto University, P.O Box 11000, 00076 Aalto, Finland; 3 Institute of Public Health and Clinical Nutrition, University of Eastern Finland, P.O. Box 1627, 70211 Kuopio, Finland; 4 Joint Municipal Authority for North Karelia Social and Health Services, Tikkamäentie 16, 80210 Joensuu, Finland; 5 Department of Public Health and Welfare, Finnish Institute for Health and Welfare, P.O. Box 30, 00271 Helsinki, Finland

**Keywords:** Real-world data, GIS, Time and travel costs, Cost model, Electronic health records

## Abstract

**Background:**

Anticoagulant therapies are used to prevent atrial fibrillation-related strokes, with warfarin and direct oral anticoagulant (DOAC) the most common. In this study, we incorporate direct health care costs, drug costs, travel costs, and lost working and leisure time costs to estimate the total costs of the two therapies.

**Methods:**

This retrospective study used individual-level patient data from 4000 atrial fibrillation (AF) patients from North Karelia, Finland. Real-world data on healthcare use was obtained from the regional patient information system and data on reimbursed travel costs from the database of the Social Insurance Institution of Finland. The costs of the therapies were estimated between June 2017 and May 2018. Using a Geographical Information System (GIS), we estimated travel time and costs for each journey related to anticoagulant therapies. We ultimately applied therapy and travel costs to a cost model to reflect real-world expenditures.

**Results:**

The costs of anticoagulant therapies were calculated from the standpoint of patient and the healthcare service when considering all costs from AF-related healthcare visits, including major complications arising from atrial fibrillation. On average, the annual cost per patient for healthcare in the form of public expenditure was higher when using DOAC therapy than warfarin therapy (average cost = € 927 vs. € 805). Additionally, the average annual cost for patients was also higher with DOAC therapy (average cost = € 406.5 vs. € 296.7). In warfarin therapy, patients had considerable more travel and time costs due the different implementation practices of therapies.

**Conclusion:**

The results indicated that DOAC therapy had higher costs over warfarin from the perspectives of the patient and healthcare service in the study area on average. Currently, the cost of the DOAC drug is the largest determinator of total therapy costs from both perspectives. Despite slightly higher costs, the patients on DOAC therapy experienced less AF-related complications during the study period.

## Introduction

Atrial fibrillation (AF) is the most common cardiac arrhythmia worldwide [1]. In Europe and the USA, it is expected that one in four middle-aged adults will be diagnosed in their lifetime [2]. The number of patients is expected to rise rapidly in the near future due to ageing populations and lifestyle factors [3, 4]. The prevalence of AF increases with age; it affects 3.7–4.2% of adults aged 60–70 and 10–17% of adults 80 years of age or older [5].

AF increases mortality, morbidity and healthcare costs, which in turn places a significant burden on healthcare systems [6]. AF is associated with a five-fold increased risk of stroke and thromboembolism [2]. Prevention of atrial fibrillation-related strokes is implemented with anticoagulants, and warfarin therapy has been the major option for decades [7]. However, the use of warfarin has not been unproblematic due to its narrow therapeutic range, the need to frequently monitor the International Normalized Ratio (INR), and the required dose adjustments [8]. In the past decade, direct oral anticoagulants (DOACs), such as dabigatran, rivaroxaban, edoxaban and apixaban, have come onto the market. DOACs are costly but easier-to-use because of fixed dosing and no need for frequent laboratory monitoring [9].

The efficacy and safety of DOACs has been compared with warfarin in randomised clinical trials. For the prevention of stroke, the results have shown that DOACs were either non-inferior to warfarin [10] or were associated with lower rates of stroke and systemic embolism [11, 12]. Both warfarin and DOAC therapies have been shown to increase a patient’s gastrointestinal bleeding risk [13, 14]. The use of DOACs has increased dramatically since their introduction onto the market. Recent studies have shown that almost 80% of new atrial fibrillation patients are started with DOACs and 20% with warfarin [9, 15]. However, there are national and regional differences regarding the use of DOACs [16–18]. Studies have also shown that with younger age persons, fewer comorbidities and a lower stroke or bleeding risk are associated with choosing DOAC over warfarin [17, 19, 20].

The total cost of anticoagulant therapies is more than the cost of the drug for the patient, especially for those receiving warfarin therapy [21]. The overall cost consists of direct and indirect costs when a patient needs frequent monitoring or travel as part of the treatment [22]. Direct costs include the costs of anticoagulant drugs and fees, which are usually treated as an out-of-pocket cost for patients. Indirect costs are caused by travel costs and the value of lost leisure time for retired persons and the loss of income for employed persons. A prior study modelled how the selection of a cost-effective anticoagulant therapy option for the patient exhibited geographic disparities when the drug, time and travel costs are taken into account [23].

Researchers have examined the costs of anticoagulation therapies using several approaches. Many cost-effectiveness studies have been done between warfarin and DOAC therapies, mostly from societal perspective [24–27]. While some studies have estimated direct costs [28, 29], few have estimated both the direct and indirect costs of warfarin therapy based on patient registry data [22, 30]. Indirect costs have often been ignored or generalised based on economic evaluations, although the cost of INR monitoring represents a large proportion of the annual cost of warfarin therapy. A recent study has estimated that almost 25% of warfarin therapy costs consist of indirect costs when patients need frequent monitoring at laboratory units [31].

However, less attention has been paid to the real-world, data-based costs of anticoagulation therapies, which cover the cost perspective of the patient and healthcare service simultaneously. Our study fills in this gap by utilising electronic health records (EHRs) and travel reimbursement data from North Karelia, in Finland, between June 2017 and May 2018 and implementing a real-world cost calculation of AF-related costs for warfarin and DOAC therapies. We evaluate the costs from the perspective of the patient and healthcare service including the direct and indirect costs of anticoagulation therapies, such as time and travel costs.

## Material and methods

### Study design

We performed a retrospective registry study over a 12-month period spanning 1 June 2017 through 31 May 2018. We retrieved information on all patients diagnosed with atrial fibrillation (ICD-10: I48) from the regional patient information system before June 2017. While AF diagnoses were not limited by any starting date, we only included those AF patients who had been on warfarin or DOAC therapy at least two months before the study period.

All AF-related costs from the perspective of the patient and healthcare service were added to the cost model based on real-world data sources and realised healthcare contacts (visits and phone consultations). We used EHRs as a source for healthcare contacts, and our data was supplemented using cost information based on healthcare visits, time and travel. For patients, time and travel costs were considered as indirect costs of anticoagulant therapies, while the other costs related to therapies were viewed as direct costs. To complete the AF-related costs of both therapies, the costs of anticoagulants were also included in the analysis. All methods were performed in accordance with the relevant guidelines and regulations.

### Study region

**Fig. 1:**
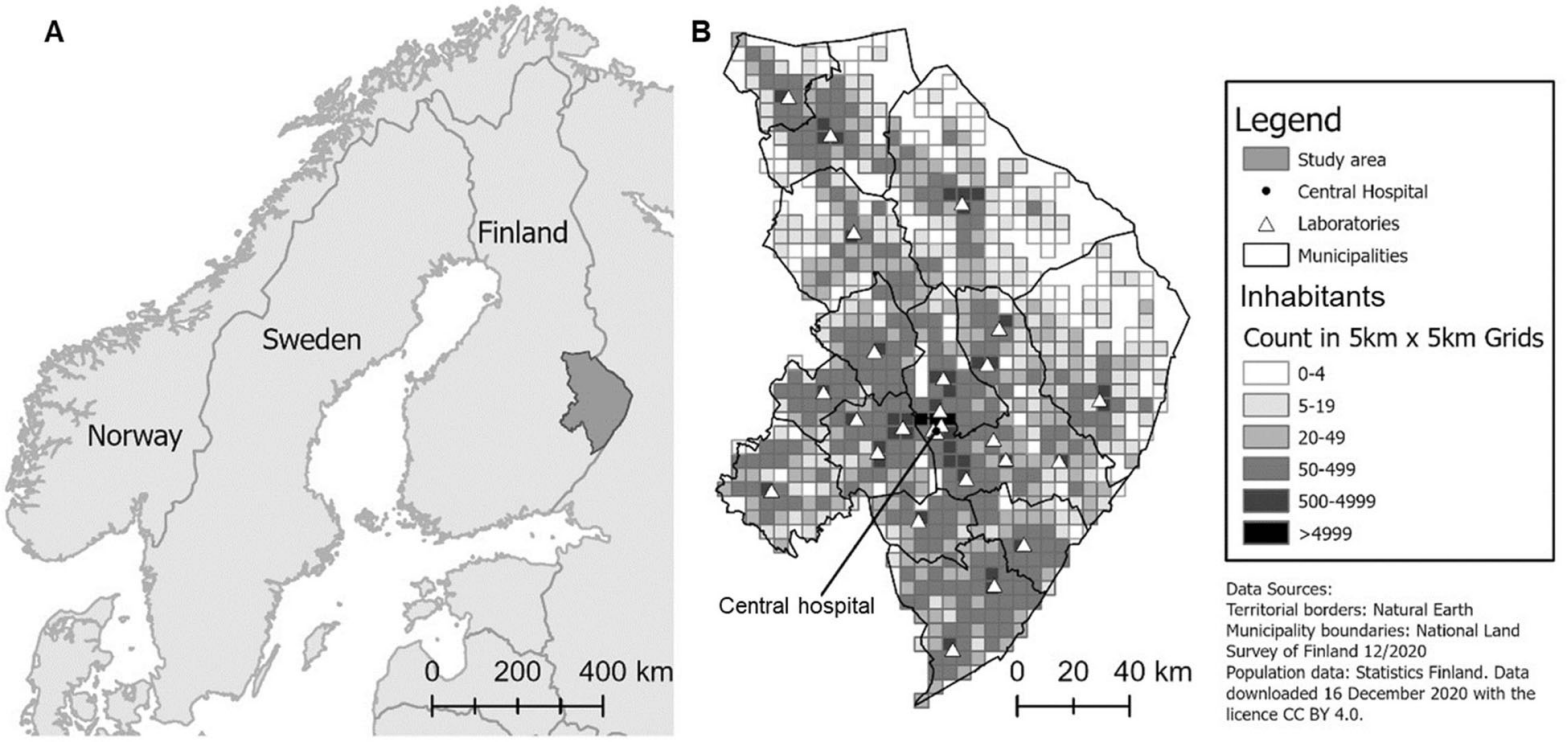
A: North Karelia, eastern Finland. B: Distribution of laboratories and inhabitants

The study region covers the healthcare district of North Karelia, Finland (the region of joint municipal authority for North Karelia social and health services, *Siun sote*). It consists of 14 municipalities, a 2017 population of 166,441 inhabitants and a population density of 8.8 persons per km^2^ [32]. Specialised healthcare services are concentrated at the central hospital, which is located in the city of Joensuu. Primary healthcare services are served by 23 public health stations in the area. INR can be monitored in 27 laboratories, which are mainly located at the health stations (Fig. 1).

### Data sources

In this retrospective registry study, we retrieved real-world data on AF patients from the regional patient information system, Mediatri. In Finland, the unique characteristic of this register is that it covers the whole healthcare district and integrates the use of primary and secondary care services. Individual-level data includes the characteristics of patients, such as age, gender, residence, the date of death, diagnoses and prescriptions. To be able to evaluate and analyse the real-world costs of AF therapies, we also collected all AF-related contacts with the healthcare, such as primary healthcare visits and phone consultations, specialised healthcare visits and phone consultations, emergency visits, laboratory results and stays on inpatient wards at the individual level. Due to a lack of cost information for the contacts, we set the real-world cost of each contact separately from the point of view of the patient and healthcare service.

We limited our analysis to only AF-related contacts. All EHRs related to primary and specialised healthcare, emergency visits and stays on inpatient wards having atrial fibrillation as primary diagnosis of the visit (ICD-10 code I48 or ICPC-2 code K78). Additionally, we considered all major complications resulting from atrial fibrillation: ischemic stroke (ICD-10 code I63), intracerebral haemorrhage (ICD-10 code I60-I62) and gastrointestinal bleeding (ICD-10 code K22, K25, K26, K27, K28, K63, K92). The EHRs for ischemic stroke and intracerebral haemorrhage were restricted to contacts that contained stays on an inpatient ward linked with an urgent visit to a healthcare. From the laboratory results, we considered all INR measurements.

We linked supplementary real-world travel information and travel reimbursement data to EHRs using the Social Insurance Institution of Finland (Kela) register, since a primary objective of our study is to obtain precise estimates for travel expenses. Kela is a government agency that provides basic economic security and national health insurance in Finland, and it reimburses part of all medical and travel costs when the patient is eligible for reimbursements [33]. We added Kela’s travel reimbursement records to EHRs if they record matched a patient’s AF-related contact. The linkage between databases was implemented using patients’ social security numbers and the dates of AF-related EHRs. The data retrieved from Kela included information about the used travel modes, the start and endpoint of taxi trips, and the travel costs. Travel expenses were given separately for patient and society in this data. Because Kela’s data covered only the part of journey information of AF-related contacts, we estimated the rest of travel costs using GIS tools, see GIS-based network analysis section.

### Selection of subjects

**Fig. 2:**
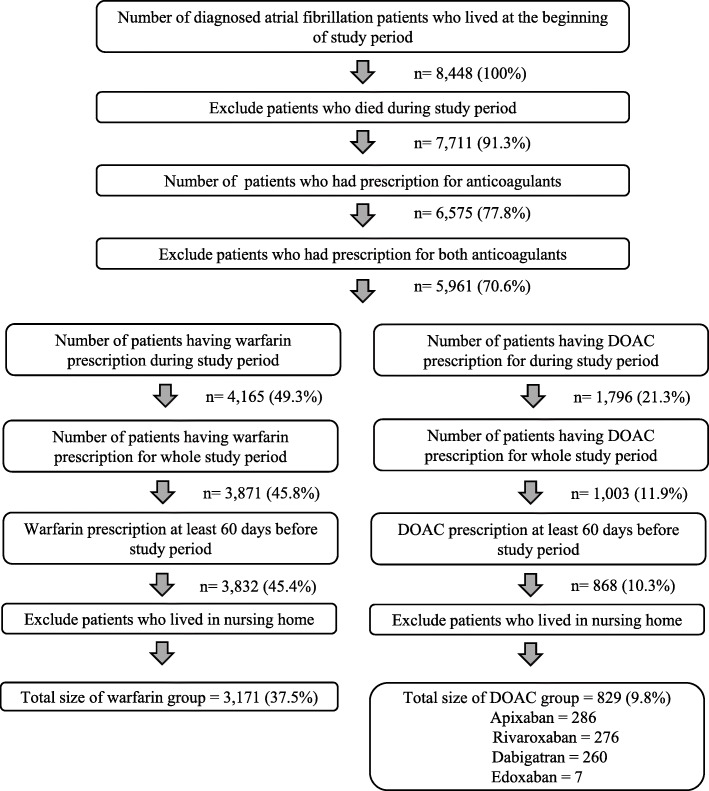
Patient attrition in the patient groups

We retrieved information on all patients from Mediatri who had received an atrial fibrillation diagnosis (*n* = 8448). Our study cohort included only AF patients who were handling the anticoagulant therapy by themselves, i.e. not living in a nursing home (Fig. 2). To be included in the study cohort, patients were required to have received a diagnosis of atrial fibrillation (ICD-10: I48) before June 2017, have a home address within the study healthcare district, still be alive at the end of the study period, have a continuous prescription for a DOAC (dabigatran, rivaroxaban, edoxaban or apixaban) or warfarin through study period and 60 days before, and be continuously receiving the same anticoagulant therapy throughout the whole study period and pre-study period. Anticoagulant therapy was considered continuous if a valid prescription was found in EHRs for the whole follow-up period.

The patients were put into different groups based on their anticoagulant therapy. The warfarin therapy study group consisted of 3171 patients, while 829 patients were in the DOAC group. Patients who had changed groups from DOAC to warfarin, or vice versa, during the period were excluded from the analysis because we wanted to calculate the real-world costs separately for warfarin and DOAC therapies based on the comparable period of time.

### Healthcare costs

We performed the cost assessment by accounting for all AF-related costs involved in primary and secondary healthcare contacts, phone consultations, emergency visits, INR monitoring, stays on inpatient wards and the outpatient medications of AF patients from the perspective of public expenditure. Secondary care costs for inpatient admissions and outpatient visits were based on the hospital’s cost accounting data. The cost of inpatient admissions in municipal wards was obtained from the national standard price list’s per diem pricing in primary healthcare multiplied by the length of stay [34]. The costs of hospitalizations included the costs of diagnostic and other types of procedures. The costs of outpatient visits and other types of contacts in primary care were based on the standard costs in the national standard price list.

The patient’s share of healthcare costs was set based on the social and healthcare customer fees in the healthcare district [35]. The payment ceiling was also considered, which limited the amount of annual fees paid by the patient to € 683. If the payment ceiling was reached during the study period, then the rest of the fees were applied to public healthcare costs.

The cost of INR monitoring was set based on the service provider information. A few INR measurements were carried out of laboratory units at the patient’s home. In these cases, we included the fixed fee for home care services for the patient as well as the visit cost related to the home care service and blood test expenses for the healthcare system.

### Drug costs

In Finland, Kela partially reimburses long-term drug treatment for chronic conditions [33]. Both warfarin and DOACs are reimbursed partially for AF patients based on the basic rate of reimbursement (40%) and the lower special rate of reimbursement (65%), respectively. The costs of anticoagulants were totalled separately for the patient and the healthcare system. The retail drug costs were shared between the patient and the healthcare service by the reimbursement percentage.

### Time and travel costs

The indirect costs of anticoagulant therapies were divided into time and travel costs in this study. These costs were determined using real-world data and geospatial modelling. The indirect costs of the patients were calculated individually for each EHR.

Time costs were set to compensate for lost working time or leisure time of patients that was spent on INR monitoring and travelling. For other healthcare contacts, we did not add time costs. We followed Jowett et al. [36] and used different weights for lost working time by age groups. The time loss for a working-age patient (< 65 years) was considered equal to the hourly gross wage, and the lost leisure time for a retired person (≥ 65 years) was valued at 35% of the average hourly gross wage. The monetary value of time to the patient was set based on the average hourly income of the postcode area data in 2017.

The travel time was calculated by the fastest route between each patient’s residential address and the location of the healthcare facility using geographic information system (GIS) based network analysis, an approach described in [37]. For car and taxi journeys, we added five minutes additional time to account for parking time and service time, respectively. The time needed to access a public transport stop was also considered in the time cost.

The EHR travel costs were determined using a combination of Kela’s register, information from the EHR and GIS-based network analysis. For each patient, travel costs were calculated as an out-of-pocket expense that they would have to pay for travelling. We included travel costs also for the healthcare services as public expenditures if the journey was reimbursed by Kela. Kela’s data showed travel costs from both perspectives for journeys in which the patient had admitted a travel allowance. However, Kela’s register covered only 5% of EHRs at time of the study period. The rest of the travel costs were calculated via a GIS-based approach.

### Travel modes and trip counts

Identifying the mode of travel is an essential component of the time and travel cost estimation. Studies have shown that multiple factors influence the choice of travel mode, such as age, gender, income, distance, physical functioning, weather, car ownership and place of residence [38, 39]. Due to a lack of travel mode information by EHRs on non-reimbursed journeys, we applied prior study classifications to set the travel modes for journeys [31, 40]. The selection criteria are shown in Table 1.

**Table 1:** The selection criteria for travel modes (the counts show realised travel mode usage based on electronic health records and travel model classification)

Travel mode	Percentage of trips	Count of one-way trips	Selection criteria	Travel speed
Private car	70	60,107 (205*)	Distance to the appointment > 1.25 km, bus not an option and patient age < 80 years or distance to the appointment > 0.25 km and patient age 80–89 years	Road speed limit
Taxi	5	4348 (1823*)	Patient age ≥ 90 years	Road speed limit
Walking	12	9913 (0*)	Distance to the appointment ≤1.25 km and patient age < 80 years, or distance ≤0.25 km and patient age 80–89 years	4 km/h
Bus	12	10,084 (8*)	Distance to the appointment > 1.25 km, destination accessible by bus, distance to the closest bus stop ≤0.25 km and patient age < 80 years	30 km/h
Ambulance	1	554 (554*)	Real-world data entries on emergency health care visits	Road speed limit
Total	100	85,069		

* Number of reimbursed trips from Kela’s database are shown in brackets

We considered five different modes of travel: car, taxi, public transport, walking and ambulance. The travel mode was conducted individually for each EHR, separately for inbound and outbound journeys. We utilised information on the distance of the journey, the age of the patient, the accessibility to public transport and the emergency classification of the healthcare visit (urgent, non-urgent) (Table 1).

We used a mixed selection of travel modes because some of the travel modes were known in advance based on Kela’s database. The mode of walking was determined following the travel mode classification scheme (Table 1). Car and public transport modes of travel were retrieved directly from Kela’s database, but in most cases the travel mode was assessed based on the travel mode classification. The mode of taxi was mostly set based on Kela’s database, but we also considered the possibility of elderly people aged 90 years and older travelling by taxi although short taxi trips under € 25 are not reimbursed and not all patients are eligible for the travel reimbursement (Table 1). The mode of ambulance was only added if the patient had an urgent visit to healthcare, if the major diagnosis was related to AF and if the travel mode information was found in Kela’s database.

A maximum one-day travel limit was set for the patients because the EHRs do not directly report a patient’s daily movements. Daily travel limits were used to exclude those journeys that most likely would not have been made due to the chaining of healthcare visits, which can be implemented at the same healthcare unit. The daily movement to a healthcare facility was limited to no more than two round-trip journeys, although there may have been more journeys based on EHR data. A more detailed classification of trip combinations and daily travel volumes are shown in Table 2.

**Table 2:** Parameters of daily trip limiters

Different visit combinations of electronic health records within a single day	Trip count
INR monitoring during inpatient ward visit	No trip
Primary or specialised healthcare visit during inpatient ward visit	No trip
First or last day at inpatient ward	One-way trip
Primary healthcare visit and first day at inpatient ward for primary healthcare	One-way trip
Specialised healthcare visit and first day at inpatient ward for specialised health care	One-way trip
INR monitoring	Roundtrip
Primary or specialised healthcare visit	Roundtrip
More than one INR monitoring at the same location	Roundtrip
INR monitoring and primary healthcare visit	Roundtrip
INR monitoring and specialised healthcare visit	Roundtrip
Primary healthcare visit, specialised healthcare visit, and first day at the inpatient ward	Roundtrip and one-way trip
One INR monitoring at health centre and another at specialised healthcare site	Two roundtrips
Primary healthcare visit, INR monitoring and specialised healthcare visit	Two roundtrips
Primary healthcare visit, more than one INR monitoring and specialised healthcare visit	Two roundtrips

### GIS-based network analysis

We used GIS to calculate the indirect time and travel costs of healthcare visits shown in the EHRs. We were able to estimate the travel time and distance of healthcare visits shown in each EHR individually. The GIS analyses also supplemented Kela’s data, since only the travel cost of the journey had been recorded in the register.

Travel analyses were implemented using ArcGIS Pro 2.5 (Esri, Redlands, CA, USA). EHRs included all essential variables, such as the address of the used healthcare facility and patient’s home address, which are compulsory source information for network analysis with an Origin-Destination (OD) cost matrix [41]. Routes to the healthcare facility were generated by combining the OD cost matrix method with the modified road network data, originally produced by the Finnish Transport Agency. We optimised routes by minimising the travel time as a good comparative measurement for the distance to health services, which has proven a successful mode of comparison at the global level [42]. The maximum travel speed was determined by the selected travel mode (Table 1). Otherwise, the speed was restricted by the speed limits for the road network.

The origin and destination with respect to the OD cost matrix was determined in two ways: (1) if the EHR contained an INR monitoring visit, the origin was the patient’s home address and the destination was the closest INR monitoring laboratory from the patient’s home; (2) if the EHR showed the patient headed to the healthcare unit, then we set the home address as the origin and the address of the healthcare unit as the destination. For INR monitoring, the closest INR laboratory was applied because the accuracy of the EHR records could not be verified in all cases. This also ensured that the cost of INR monitoring is within reason.

Travel cost was calculated based on the information in the EHR, if the journey was not included in Kela’s data. The calculation was based on travel distance and fares. We created travel cost equations for each travel mode applying Ford et al. [40] and Leminen et al. [31]. The travel cost can be calculated with eqs. (1–4) for private car (*c*_c_), taxi (*c*_x_), walking (*c*_w_) and public transport (*c*_b_):

1 $$ {c}_{\mathrm{c}}={o}_{\mathrm{c}}d $$

2 $$ {c}_x={f}_{\mathrm{t}}+{o}_{\mathrm{t}}d $$

3 $$ {c}_w=0 $$

4 $$ {c}_b={f}_b $$

where d is the distance of the journey, *o*_c_ is the operation cost of the car per kilometre, *f*_t_ is the fixed charge of the taxi, *o*_t_ is the operation cost of the taxi per kilometre and *f*_b_ is the public transport fare. We did not add any travel cost for walking. The values of the parameters are presented in Table 3.

**Table 3:** Summary of parameters

Parameter	Description of parameters	Value
Fixed parameters		
*t*_m_	Time loss associated with INR monitoring	0.5 h
*t*_a_	Parking time for car or service time for taxi	0.083 h
*c*_x_	Daily cost of anticoagulant medicine for patient	Warfarin = € 0.08, DOACs = € 0.95–1.00
*v*_p_	Cost of primary healthcare visits for patient	€ 11.4 or € 20.6
v_s_	Cost of specialised healthcare visits for patient	€ 41.2
*d*_p_	Cost of primary healthcare inpatient ward	€ 48.9
*d*_s_	Cost of specialised healthcare inpatient ward	€ 48.9
*o*_c_	Operation cost of car per kilometre	€ 0.43 / km
*o*_t_	Operation cost of taxi per kilometre	€ 1.59 / km
*f*_b_	Public transport fare	€ 2.00, 3.80 or 5.00
*f*_t_	Fixed charge for taxi	€ 5.9
*s*	Size of patient group	Warfarin = 3171, DOAC = 829
*d*_h_	Daily medicine cost for healthcare	Warfarin = € 0.05, DOACs = € 1.76 or 1.87
*i*_s_	Cost of INR monitoring for healthcare per visits	€ 31 (blood test + healthcare personnel cost)
*p*_a_	Patient’s hourly gross wage coefficient	Working time is valued as 100% (age < =65) and leisure time as 35% of the hourly wage (age > 65)
Real-world data parameters		
*m*	INR monitoring count	Based on EHR
*e*_p_	Cost of primary care visits for healthcare	Based on EHR
*e*_s_	Cost of specialised healthcare visits for healthcare	Based on EHR
*w*_p_	Cost of inpatient ward for primary care	Based on EHR
*w*_s_	Cost of inpatient ward for specialised healthcare	Based on EHR
*c*_t_	Patient’s time cost per hour	From Zip code area income data
*t*_t_	Total travelling time	Calculated using network analysis
*d*	Distance of journey	Calculated using network analysis
*t*_j_	Travel time of journey	Calculated using network analysis
*c*_e_	Travel cost for ambulance	Kela database
*k*	Cost of travel reimbursement for healthcare	Kela database

The time cost for the journey was calculated based on the travel time and additional time penalties. The time cost can be calculated with eqs. (5–6) for private car (T_c_), taxi (T_x_), walking (T_w_), public transport (T_b_) and ambulance (T_e_):

5 $$ {T}_{\mathrm{c}=\mathrm{x}}={p}_{\mathrm{a}}{c}_{\mathrm{t}}\ {t}_{\mathrm{j}}+{t}_{\mathrm{a}} $$

6 $$ {T}_{\mathrm{w}=\mathrm{b}=\mathrm{e}}={p}_{\mathrm{a}}{c}_{\mathrm{t}}{t}_{\mathrm{j}} $$

where *t*_j_ is the travel time for the journey, *p*_a_ is the patient’s hourly gross wage coefficient, *c*_t_ is the patient’s time cost per hour and *t*_a_ is the parking time for the car or service time for the taxi.

### Cost model

The cost model was created to calculate the real-world costs of anticoagulant therapies from the perspective of the patient and healthcare service. We applied prior studies by Leminen et al. (2019) and Pyykönen et al. (2019), who have explored the cost impacts of medication selection for AF patients. In earlier studies, the costs of anticoagulant therapies have focused on a combination of INR monitoring and anticoagulant medication. We broadened the cost model to cover all AF-related healthcare costs from both perspectives (patient’s out-of-pocket cost and healthcare public expenditure). In addition to previous models, we specified daily travel limits (Table 2) and used travel reimbursement data from Kela to supplement the calculation of indirect costs.

**Fig. 3:**
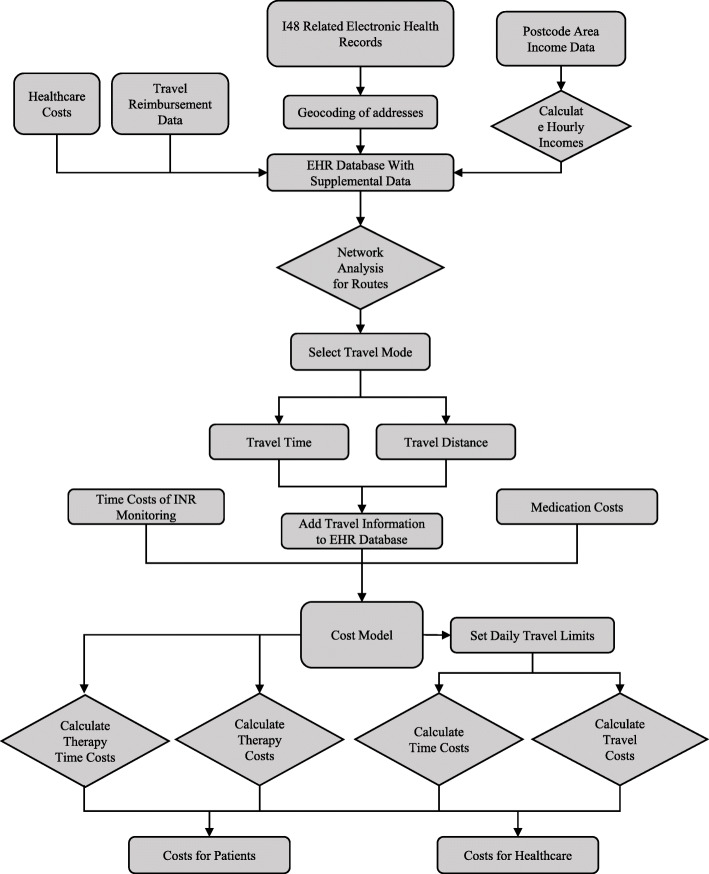
Flowchart of the cost calculation for electronic health records

The structure of the cost model is also shown in Fig. 3. The costs are calculated separately from the perspective of patients and the healthcare service. From the patients’ perspective, the costs can be calculated with eqs. 7–12. We included travel costs for healthcare based on five different travel modes (Table 1), the time costs associated with frequent INR monitoring and travelling, medication costs based on the costs of anticoagulants and healthcare fees based on EHRs. These costs together comprised the AF-related, real-world costs from the patient perspective.

The total costs (${\mathrm{c}}_{\mathsf{p}, id}$) for *i*^th^ patient can be defined on day *d* as follows:

7 $$ {c}_{\mathrm{p}, id}={c}_{\mathrm{m}, id}+{c}_{\mathrm{a}, id}+{c}_{\mathrm{t}, id}+{c}_{\mathrm{d}, id} $$

where ${c}_{\mathsf{m}, id}$ is the time cost for anticoagulation management (€), ${c}_{\mathrm{a}, id}$ is the direct cost of the anticoagulant therapy (€), ${c}_{\mathsf{t}, id}$ is the time cost for travelling (€) and ${c}_{\mathsf{d}, id}$ is the travel cost (€).

The time cost of anticoagulation management (${c}_{\mathsf{m}, id}$) for *i*^th^ patient on day *d* is calculated as follows:

8 $$ {c}_{\mathrm{m}, id}={m}_{id}{c}_{\mathrm{h},i}{p}_{\mathrm{a},i}{t}_{\mathrm{m}} $$

where ${m}_{id}$ is the count of INR monitoring visits, ${c}_{\mathsf{h},i}$ is the patient’s time cost per hour (€), ${p}_{\mathsf{a},i}$ is the hourly gross wage coefficient and *t*_m_ is the time used for INR monitoring (h) per visit.

The direct cost of anticoagulant therapy (${c}_{\mathsf{a}, id}$) for *i*^th^ patient on day *d* can be defined as:

9 $$ {c}_{\mathrm{a}, id}={c}_{\mathrm{x},d}+{v}_{\mathrm{p}, id}+{v}_{\mathrm{s}, id}+{d}_{\mathrm{p}, id}+{d}_{\mathrm{s}, id} $$

with ${c}_{\mathsf{x},d}$ the daily cost of anticoagulant medicine for patient (€), ${v}_{\mathsf{p}, id}$ the cost of primary healthcare visits (€), ${v}_{\mathsf{s}, id}$ the cost of specialised healthcare visits (€), ${d}_{\mathsf{p}, id}$ the cost of the primary healthcare inpatient ward (€), and ${d}_{\mathsf{s}, id}$ the cost of a specialised healthcare stay on the inpatient ward (€).

The time cost of travelling (${c}_{\mathsf{t}, id}$) for *i*^th^ patient on day *d* is calculated as follows:

10 $$ {c}_{\mathrm{t}, id}={c}_{\mathrm{h},i}{p}_{\mathrm{a},i}{t}_{\mathrm{t}, id} $$

where ${c}_{\mathsf{h},i}$ is the patient’s time cost per hour (€), ${p}_{\mathsf{a},i}$ is the patient’s hourly gross wage coefficient and ${t}_{\mathsf{t}, id}$ is the total travelling time in hours based on network analysis for day *d*.

The travel cost (${c}_{\mathsf{d}, id}$) for *i*^th^ patient on day *d* is then estimated as follows:

11 $$ {c}_{\mathrm{d}, id}={c}_{\mathrm{c}, id}+{c}_{\mathrm{x}, id}+{c}_{\mathrm{b}, id}+{c}_{\mathrm{e}, id} $$

where ${c}_{\mathsf{c}, id}$ is the travel cost for the car, ${c}_{\mathsf{x}, id}$ is the travel cost for the taxi, ${c}_{\mathsf{b}, id}$ is the travel cost of public transport and ${c}_{\mathsf{e}, id}$ is the travel cost for the ambulance.

From the patients’ perspective, the averaged real-world cost of anticoagulation therapy (warfarin or DOAC) per patient (${r}_{\mathsf{p}}$) can be solved with the following equation:

12 $$ {r}_{\mathrm{p}}={\sum}_i{\sum}_d\frac{\left({c}_{\mathrm{m}, id}+{c}_{\mathrm{a}, id}+{c}_{\mathrm{t}, id}+{c}_{\mathrm{d}, id}\right)}{s} $$

where ${r}_{\mathsf{p}}$ is the average cost for the patient group during the follow-up period and *s* is the size of the patient group.

From the perspective of the healthcare service, the real-world costs consist of primary and specialised healthcare contacts, stays on inpatient wards, medication costs, INR monitoring and travel reimbursement costs for society. The averaged real-world healthcare costs per patient (${r}_{\mathsf{h}}$) can be defined for both anticoagulation therapies as follows:

13 $$ {r}_{\mathrm{h}}={\sum}_i{\sum}_d\frac{\left({e}_{\mathrm{p}, id}+{e}_{\mathrm{s}, id}+{w}_{\mathrm{p}, id}+{w}_{\mathrm{s}, id}+{d}_{\mathrm{h}, id}+{k}_{id}+\left({m}_{id}\ast {i}_{\mathrm{s}, id}\right)\right)}{s} $$

where ${e}_{\mathsf{p}, id}$ is the cost of primary healthcare visits for healthcare, ${e}_{\mathsf{s}, id}$ is the cost of specialised healthcare visits for healthcare, ${w}_{\mathsf{p}, id}$ is the cost of the inpatient ward for primary healthcare at the healthcare facility, ${w}_{\mathsf{s}, id}$ is the cost of the inpatient ward for specialised healthcare at the healthcare facility, ${d}_{\mathsf{h}, id}$ is the medicine cost for the healthcare facility, ${k}_{, id}$ is the travel reimbursement cost for society based on Kela’s data, ${m}_{, id}$ is the cost of INR monitoring visits and ${i}_{\mathsf{s}, id}$ is the INR monitoring cost for the healthcare service (including the cost of blood test and healthcare personnel costs).

## Results

### Characteristics of the patient groups

The total study population consisted of 4000 atrial fibrillation patients after the inclusion and exclusion criteria were applied. The key characteristics of the patient groups are summarised in Table 4. Most AF patients who were taking anticoagulants were in the warfarin group (*n* = 3171). The DOAC group consisted of 829 patients. The mean age of the patients was higher in the warfarin group compared to the DOAC group (76.0 ± 9.2 vs. 72.7 ± 9.8), respectively. The CHA2DS2-VASc score for stroke risk assessment was almost equal in both groups. The most prevalent co-morbidity was hypertension, followed by vascular disease and diabetes in both groups. Females were in the minority in both groups.

**Table 4:** Key characteristics of the study population

Variable	Warfarin	DOAC
N	3171	829
Age, mean ± S.D.	76.0 ± 9.2	72.7 ± 9.8
Female gender, no. (%)	1485 (46.8)	391 (47.2)
CHA2DS2-VASc score, mean ± S.D.	3.2 ± 1.5	3.0 ± 1.6
Diabetes, no. (%)	862 (27.2)	217 (26.2)
Hypertension, no. (%)	1338 (42.2)	414 (49.9)
Vascular disease, no. (%)	876 (27.6)	207 (25.0)
Congestive heart failure, no. (%)	522 (16.5)	107 (12.9)
Transient ischemic attack (TIA), no. (%)	113 (3.6)	51 (6.2)
Time in Therapeutic Range (TTR), mean ± S.D.	78.3 ± 19.1	
AF-RELATED COMPLICATIONS DURING STUDY PERIOD		
Intracerebral haemorrhage, no. (%)	11 (0.3)	0 (0.0)
Gastrointestinal bleeding, no. (%)	76 (2.4)	7 (0.8)
Ischemic stroke, no. (%)	16 (0.5)	4 (0.5)
HEALTHCARE UTILISATION RELATED TO AF		
AF-related visits to healthcare site, mean ± S.D.	18.0 ± 10.2	0.7 ± 1.5
Travelling count by the patient, mean ± S.D.	13.2 ± 8.0	0.6 ± 1.3
Number of all INR measurements, mean ± S.D.	17.5 ± 9.9	0.1 ± 0.9
Number of INR measurements during hospitalisation, mean ± S.D.	2.5 ± 6.2	0.1 ± 0.3
Primary healthcare visits, mean ± S.D.	0.2 ± 0.8	0.2 ± 0.8
Days on ward for primary healthcare, mean ± S.D.	0.2 ± 2.7	0.2 ± 2.4
Specialised healthcare visits, mean ± S.D.	0.2 ± 0.7	0.3 ± 0.9
Days on ward for specialised healthcare, mean ± S.D.	0.1 ± 1.0	0.2 ± 1.2
GEOGRAPHICAL CHARACTERISTICS		
Distance to the closest laboratory (km), mean ± S.D.	5.5 ± 7.6	5.6 ± 7.3
Distance to the closest primary healthcare centre (km), mean ± S.D.	6.0 ± 7.8	6.4 ± 7.6
Distance to specialised healthcare services (km), mean ± S.D.	52.1 ± 42.1	43.9 ± 40.0
Urban residence, no. (%)	2292 (72.3)	568 (68.5)
Postal code area income (€), mean ± S.D.	12.0 ± 1.0	12.3 ± 1.2

The occurrence of intracerebral haemorrhage and gastrointestinal bleeding during the study period was higher in the warfarin group than the DOAC group (Table 4). Only a few events of ischemic stroke occurred during the study period, and the occurrence rate was equal in both groups.

### Healthcare resource use

The total number of AF-related visits to healthcare facilities varied significantly among the patient groups (Table 4). On average, patients receiving warfarin therapy had 18.0 ± 10.2 AF-related visits to healthcare facilities, compared to only 0.7 ± 1.5 visits for those receiving DOAC therapy (incl. for primary and specialised healthcare, inpatient wards and INR monitoring) during the one-year study period. When the maximum count of daily trips was applied in the model, patients travelled to healthcare facilities on average 13.2 ± 8.0 times when receiving warfarin therapy and 0.6 ± 1.3 times when receiving DOAC therapy.

The number of visits to primary healthcare, specialised healthcare and inpatient wards did not differ between the warfarin and DOAC groups, with only minor differences detected. On average, specialised healthcare visit counts (0.2 ± 0.7 vs. 0.3 ± 0.9) and days on the ward for specialised healthcare (0.1 ± 1.0 vs. 0.2 ± 1.2) were slightly higher for the DOAC group.

### Geographical access to care

Travel impedance for both patient groups is reported in Table 4. In both groups, most patients lived in urban areas. The share of those with an urban residence was slightly lower in the DOAC group (68.5% vs. 72.3%). The average travel distance to the nearest laboratory using the road network was 5.5 km for those in the warfarin group. On average, the distance to the primary and specialised healthcare wards was 6.0 and 52.1 km, respectively. The travel distances for those in the DOAC group closely matched the distances for those in the warfarin group. Only the travel distance to the specialised healthcare (52.1 km ± 42.1 vs. 43.9 km ± 40.0) was clearly lower for those in the DOAC group.

### Real-world costs of therapies

AF-related real-world costs during the one-year follow-up period for those in the warfarin and DOAC groups is displayed in Table 5. On average, the cost per patient for healthcare as public expenditure was higher on DOAC therapy (average ± S.D. cost = € 927 ± € 1150.9 vs. € 805 ± € 1118.7). Additionally, the average cost of anticoagulant therapy for patients was also higher for those receiving DOAC therapy (average ± S.D. cost = € 406.5 ± € 132.8 vs. € 296.7 ± € 256.3).

**Table 5:** Atrial fibrillation-related real-world costs (€) of anticoagulation therapies during the one-year follow-up period. The CIs for the cost difference were based normal distributions

Type of Cost	Warfarin mean ± S.D.	95% CI	DOAC mean ± S.D.	95% CI	Cost difference (Warf - DOAC)	*p*-value
AF-related total cost for healthcare	805.2 ± 1118.7	766.3–844.1	927.3 ± 1150.9	849.0–1005.6	− 122.1	0.006
AF-related total cost for patient	296.7 ± 256.3	287.8–305.6	406.5 ± 132.8	397.5–415.5	−109.8	< 0.001
AF-related total cost (patient + healthcare)	1102.0 ± 1272.2	1057.7–1146.3	1333.8 ± 1250.8	1248.7–1418.9	−231.8	< 0.001
Patient costs						
Patient’s time and travel cost	195.6 ± 221.8	187.9–203.3	17.5 ± 61.5	13.3–21.7	178.1	< 0.001
Patient’s time cost	79.5 ± 84.4	76.6–82.4	3.3 ± 11.2	2.5–4.1	76.2	< 0.001
Patient’s travel cost	118.2 ± 168.1	112.3–124.1	14.3 ± 52.4	10.7–17.9	103.9	< 0.001
Healthcare fees for patient	74.0 ± 155.0	68.6–79.4	29.2 ± 88.0	23.2–35.2	44.8	< 0.001
Drug cost for patient	27.0 ± 0.0	27.0–27.0	359.8 ± 10.4	359.1–360.5	− 332.8	< 0.001
Healthcare costs						
INR monitoring cost	519.2 ± 296.2	508.9–529.5	4.3 ± 14.1	3.3–5.3	514.9	< 0.001
Primary healthcare visit costs	12.7 ± 51.7	10.9–14.5	16.6 ± 47.5	13.4–19.8	−3.9	0.042
Specialised healthcare visit costs	61.1 ± 303.9	50.5–71.7	100.3 ± 608.5	58.9–141.7	−39.2	0.072
Ward costs in primary healthcare	12.2 ± 130.2	10.9–14.5	13.9 ± 122.4	5.6–22.2	−1.7	0.731
Ward costs in specialised healthcare	78.6 ± 706.7	54.0–103.2	88.4 ± 781.0	35.2–141.6	−9.8	0.743
Drug cost for society	18.0 ± 0.0	18.0–18.0	668.2 ± 19.4	666.9–669.5	− 650.2	< 0.001
Kela’s travel cost for society	99.3 ± 305.4	88.7–109.9	26.5 ± 110.8	19.0–34.0	72.8	< 0.001

With respect to the real-world cost per patient for healthcare, we included AF-related laboratory monitoring (INR), primary and specialised healthcare visits, phone consultations, stays on inpatient wards, anticoagulation costs, travel costs compensations and INR monitoring provided at the residence of the patient. The largest share of the costs for healthcare was caused by INR monitoring for those receiving warfarin therapy (€ 519.2, 64.5%) and drug costs for those receiving DOAC therapy (€ 668.2, 72.1%), on average (Table 5).

From the patients’ perspective, the real-world cost of anticoagulation therapy consisted of time costs, travel costs, healthcare fees and drug costs. For those receiving warfarin therapy, the combination of time and travel costs accounted for 65.9% of the total costs, on average, due to frequent INR monitoring and relatively affordable warfarin medication (Table 5). Respectively, time and travel costs totalled only 4.3% for those receiving DOAC therapy, and the largest share for the patient was the drug costs, with the share being 88.5%. For those receiving warfarin therapy, the costs varied more among patients than for those receiving DOAC therapy (Table 5). Many variables, such as travel mode, travel distance and the lost working and leisure time of the patient, resulted in a range of the total costs for those receiving warfarin therapy from the patient perspective.

The costs of anticoagulation therapies include both direct and indirect costs for patients. Of the annual costs for those receiving warfarin therapy, 66.2% (€ 195.6) had to do with indirect time and travel costs and 33.8% (€ 101.1) with direct costs (drugs and fees), on average. Respectively, only 4.3% (€ 17.5) of costs for those receiving DOAC therapy were indirect costs, and direct costs accounted for 95.7% (€ 389.0) of expenses, on average. Due to the sparsely populated area and frequent INR monitoring of those receiving warfarin therapy, the share of indirect costs was relatively high for those receiving warfarin therapy compared with the DOAC therapy group.

## Discussion

We gathered a wide array of retrospective, real-world data on atrial fibrillation patients, data which consisted of 4000 patients from North Karelia, Finland. We found marked differences between the annual costs of anticoagulation therapies from the perspectives of the healthcare service and patients. When we included all AF-related, real-world costs of warfarin and DOAC therapies, including the most common complications, the average costs for those receiving warfarin therapy were lower from the perspectives of the healthcare service (€ 927 vs. € 805) and patient (€ 406.5 vs. € 296.7) in the study area, respectively. However, patients receiving the warfarin therapy experienced more AF-related complications (intracerebral haemorrhage and gastrointestinal bleeding) during the study period.

The main reason for the higher annual costs for those receiving DOAC therapy currently is the cost of the DOAC drug. However, DOAC drugs eliminate the overall costs for medical consultation, laboratory tests, transportation and the time loss of the patient. On average, the share of drug costs for those receiving DOAC therapy was 88.5% for the patient and 72.1% per patient from the perspective of the healthcare public expenditure in our study settings, meaning that the cost of the DOAC drug primarily determines the cost of the DOAC therapy from both perspectives, but it does not place a burden on healthcare service use. Respectively, INR monitoring accounts for the largest share of warfarin therapy costs (64.5%) per patient for the healthcare service, on average, and the combination of time and travel costs (65.9%) for patients receiving warfarin therapy. In the case of warfarin therapy, INR monitoring induces a burden for healthcare services and travelling causes additional indirect therapy costs for the patient.

Although the real-world costs of the DOAC therapy were higher on average, the annual costs can be lower for patients, especially in distant localities. It is important to notice that time and travel costs can significantly contribute to the annual costs of anticoagulation therapies, especially for those receiving warfarin therapy. The annual travel costs for the patient varied from € 0 to € 1888.8 in the study area, depending on the patient’s visits for warfarin therapy. The real-world cost difference between the anticoagulation therapies is not big when the geographical characteristics of the therapies are considered in the cost model. A prior study has shown that the cost-efficiency of the anticoagulation therapy option for the patient depends on travel distance, travel mode, drug costs and the lost working and leisure time of the patient [23]. In urban areas, where healthcare facilities are easily accessible, warfarin therapy is usually the least-cost option. When travel distance to sample collection points increases, then the more expensive DOAC drug can be cost-efficient for the patient if drug, time and travel costs for the warfarin therapy exceed the costs of the DOAC medication.

The key strength of this study is the inclusion to several datasets for modeling. Firstly, our data covers all patients having AF diagnosis in the study area. Secondly, our dataset is very rich in terms of information on health care use. Used EHRs and additional data from Kela formulate vast and precise data for the analyses. Thirdly, in addition to direct health care costs, we have been able to consider a wide range of actual other costs based on various register data sources.

The study suffers from a few limitations. First, we managed to find information on travel modes from real-world data, but most travel modes had to be set based on the travel mode classification due the lack of travel mode data. This might have a slight effect on travel mode selection for shorter journeys, but the effect on costs is minimal. Second, we considered only the costs of AF-related events when the payment ceiling for healthcare costs was applied. The payment ceiling could limit healthcare costs for the patient more if all-cause healthcare costs would be included in the study. Third, we did not consider the variation in warfarin drug consumption. The annual cost of the warfarin drug was calculated using the average dosage for all patients, thus the annual cost of the warfarin drug might in reality be higher for some patients. In this case, the annual difference between therapies would be narrowed. Fourth, our data was gathered from one healthcare district which represents only a small part of Finland. This might affect to representativeness of results at the level of whole Finland. Fifth, the costs of warfarin therapy are comparable to other studies when the costs are compared after the initiation period of administering the warfarin drug. We did not include patients who had started taking the anticoagulant medication just before or during the study period. The average annual costs for warfarin therapy would be higher due to more frequent monitoring of INR during the initiation phase of taking warfarin.

We had to set most travel modes based on the travel mode classification. Despite a lack of real-world travel mode data, our travel mode classification is in line with findings presented in a prior study, which had collected multinational, real-world data on travel modes when patients were attending INR monitoring [34]. The shares of travel mode usage seem to match our classification almost identically when compared to Sweden and, for the most part, France and Australia.

We did not include the lost time spent in hospital as hospitalization is not a typical process in performing the therapy. Hospitalization is related to complications and there are various other factors, which are hard to control and which affects to the length of the stay. In addition, the number of days on wards was very small and similar in both treatment groups.

The patients in the DOAC group had a lower mean age (72.7 vs. 76.0) and experienced less gastrointestinal bleeding and intracerebral haemorrhage events. In addition to patients treated with warfarin being at greater risk for these complications [11, 12], also the younger age of DOAC patients might have slightly affected the difference. It seems that DOAC treatment is more often selected as a form of treatment for younger patients, as has been observed also in previous studies [19]. However, the difference in treatment-related complications between the patient groups was relatively small, indicating that the warfarin therapy in North Karelia is reasonably well managed.

The maximum one-day travel limit was applied when time and travel costs were calculated. If the travel costs for all AF-related trips recorded in the EHRs (average ± S.D., warfarin group = 18.0 ± 10.2, DOAC group = 0.7 ± 1.5) would be included in the cost model, time and travel costs would be higher for those receiving warfarin therapy. In that case, the cost difference between anticoagulation therapies would even be narrowed and the annual average costs of anticoagulation therapies would be almost equal from the patients’ perspective. A prior study has reported that patients receiving warfarin therapy have 17.0 INR monitoring visits per year on average [27]. We added time and travel costs for 13.2 visits by patients on warfarin therapy, on average, including other AF-related journeys to healthcare facilities. Since we only considered the realised journeys and the possibility of chaining healthcare visits for one day, our results show the baseline of time and travel costs for anticoagulation therapies.

The costs of drugs, hospitalisations, healthcare visits and travel vary between countries. If the results of this study are compared with other real-world cost studies, these aspects should be taken into account. We also considered reimbursements for drugs and travel and the payment ceiling for the patient based on guidelines from the Social Insurance Institution of Finland. This will have an influence on the observed costs and must be considered when comparing results between countries. In addition to healthcare service cost differences, it is important to note that populations density is quite low in the study area (8.8 per km2). This affects patients’ indirect costs because travel distances can be relatively long for patients in rural areas.

The patients in the DOAC group seemed to live closer to the specialised healthcare services (43.9 km ± 40.0 vs. 52.1 km ± 42.1) than patients in the warfarin group. Specialised healthcare services are only served in the provincial centre, Joensuu. This might increase the indirect costs of warfarin therapy, but patients in both groups had only a few visits to specialised healthcare facilities during the study period.

The cost model can be applied for different diseases when at least one therapy includes time and travel costs. The use of EHRs is common in health service research, and a few studies have already combined GIS methods to supplement collected real-world data [31, 37, 43, 44]. The strength of GIS methods is especially evident when information about patients’ travelling costs is required in large areas. The comparison can be implemented by changing the main parameters and applying real-world data sources. No matter, the treatment carried out was similar in the case of anticoagulant therapies or when using remote monitoring vs. laboratory monitoring.

## Conclusion

Due to high drug costs for DOACs, the DOAC therapy had higher total costs than warfarin in the healthcare district but the cost difference between therapies was otherwise narrow in our study setting. However, the causes of the costs are different due to dissimilar implementation practices between the anticoagulation therapies. It is crucial to include additional indirect costs, such as time and travel costs associated with warfarin therapy, due to their distinct effect on the anticoagulation therapy cost. The results suggest that indirect costs should be included in economic evaluations when comparing costs and cost-effectiveness between anticoagulation therapies in society.

## Data Availability

The data that support the findings of this study are available from joint municipal authority for North Karelia social and health services but restrictions apply to the availability of these data, which were used under license for the current study, and so are not publicly available. Data are however available from the authors upon reasonable request and with permission of joint municipal authority for North Karelia social and health services.

## Abbreviations

AF: Atrial fibrillation
DOAC: Direct oral anticoagulant
EHR: Electronic health record
GIS: Geographic Information System
INR: international normalised ratio
Kela: Social Insurance Institution of Finland
